# High gelatinous salted duck egg white protein powder gel: Physicochemical, microstructure and techno-functional properties

**DOI:** 10.3389/fnut.2023.1110786

**Published:** 2023-02-03

**Authors:** Xinjun Yao, Jicheng Xu, Yu Xun, Tianyin Du, Mengqi Huang, Jun Guo

**Affiliations:** ^1^College of Biological and Food Engineering, Anhui Polytechnic University, Wuhu, China; ^2^College of Biology and Food Science, Suzhou University, Suzhou, China

**Keywords:** salted duck egg white, protein peptide powder, high gelatinous, physicochemical properties, xanthan gum

## Abstract

Salted duck egg is one of the most popular products, and China is one of the major countries consuming salted duck egg products. However, due to the high salt content of salted egg white and low physical and chemical properties such as gel, many factories generally only use salted egg yolk and discard salted duck egg white (SDEW) as a waste liquid when processing. This is not only a waste of resources, but also a pollution to the environment. In this paper, protein powder was prepared from salted egg white. Then xanthan gum (XG) was added to make it co-gel with ovalbumin to achieve the purpose of preparing high gelatinous salted egg white protein powder. The results showed that the optimum conditions of SDEW-XG composite gel were as follows: the xanthan gum content was 0.08% (w/w), the reaction pH was 6.5, and the heating temperature was 100°C. Under these conditions, the gel strength reaches the maximum value. Meanwhile, compared with the protein powder without xanthan gum, the addition of xanthan gum significantly affected the secondary structure of the protein powder of SDEW and improved the water holding capacity of the gel. In conclusion, the addition of xanthan gum can significantly improve the gel quality of SDEW protein powder, which provides a theoretical basis for the quality improvement of salted egg white.

## Introduction

Salted duck eggs are a traditional food in China and South Asia. The salted duck egg white (SDEW) makes up 50 to 60% of the whole salted duck egg (1–3). Salted duck egg in the production process, involves the step of pickling. Pickling does not change the composition of the protein, but it does add a lot of salt. High concentration of NaCl changed the gel performance of SDEW, which would greatly reduce the utilization rate of SDEW (4–6). Currently, only a small portion of SDEW is used in animal feed, and most of it is discarded. So far, many scholars have studied the reuse of SDEW (7–9). Peng et al. (10) prepared a novel composite protein membrane by cross-linking egg white protein with the Transglutaminase enzyme. The study showed that the protein membrane containing the Transglutaminase enzyme was more uniform, smooth, and had better water resistance and thermal stability. Tang et al. (11) investigated the effect of carrageenan on the protein structure and gel properties of SDEW. The results showed that the addition of carrageenan can significantly increase the content of the free sulfhydryl group and surface hydrophobicity of SDEW protein. At the same time, with the increase of carrageenan content, the hardness, viscosity, chewability, and elasticity of the gel prepared from SDEW were improved.

The production of high gelatinous egg white protein powder in China is very low now, mainly the general protein powder. Therefore, a large number of scholars have studied the preparation of high gelatinous egg white protein powder (12–14). However, most of these studies take egg white as the research object, and it is still rare to use SDEW as raw material to prepare high-quality protein powder. This is mainly because the high concentration of NaCl in SDEW seriously affects its functional properties such as gelation and water holding capacity (15). So, a large amount of salted egg white is discarded, resulting in a waste of high-quality protein resources. At the same time, it also causes pressure to the environment to a certain extent.

When egg white is used as gelling agent in food processing, the addition amount is relatively high and the production cost is high. Although ordinary egg white powder is more hygienic and convenient than fresh egg white, its gel properties are often inferior to fresh egg white. So further systematic research on the gel properties of egg white and the modification of egg white powder has high practical value. The scheme of this research just provided a feasible way to solve this problem. In this research, SDEW was used as raw material, and after desalting, it was mixed with a certain amount of xanthan gum to make the two co-gels to obtain high gelatinous SDEW protein powder. Then, the water holding capacity and gel microstructure of high gelatinous SDEW protein powder were studied. This study provided a new idea for the comprehensive utilization of SDEW. In a sense, it can effectively reduce the waste of resources and environmental pollution.

## Materials and methods

### Raw materials

SDEW was provided by Anhui Tianhe Food Co., LTD. SDEW was placed in the refrigerator at −20°C and thawed at room temperature before use. Xanthan gum was purchased from Hefei Pomeranian Biotechnology Co., LTD.

### Preparation method of protein powder

SDEW liquid was filtered with four layers of gauze to remove impurities (eggshell and small stones). Then it was diluted five times and the salt content was 9.3% by salinometer. Then, in order to remove salt, the SDEW was ultrafiltered to obtain the final desalted egg white with a salt content of 0.63%. The desalted SDEW liquid was adjusted with 40% citric acid to pH 5.5. Then 0.2% dry yeast was heated in a water bath at 30°C for 4 h. The SDEW liquid was spray dried (feed temperature 180 ± 3°C, discharge temperature 70 ± 3°C) with a spray dryer (B-290, BÜCHI Labortechnik AG, Flawil, Switzerland) to obtain protein powder.

### Preparation method of gel

The protein powder was prepared into a 10% protein powder solution. Xanthan gum was added to the protein powder solution and the pH of the solution was adjusted to 6.5 (40% citric acid, 2 mol/L NaOH). The solution was stirred with a magnetic stirrer (MS123D, Showrange Ltd., Shanghai, China) at a speed of 120 r/min for 5 min. The beaker was sealed with plastic wrap. After heating in a water bath for 1 h (100°C), the beaker was immediately cooled in an ice-cold water bath for 20 min. Then, the beaker was placed in the refrigerator (BCD-485WSPZM, Meidi Ltd., Guangzhou, China) at 4°C for 24 h to make the gel.

### Optimization of experimental conditions using the single-factor test

#### Single factor test of xanthan gum addition

Under the conditions of pH 6.5, heating temperature 100°C, and heating time 1 h, six groups of xanthan gum contents (0, 0.04, 0.08, 0.12, 0.16, and 0.20%) were selected as the research objects. The objective of this experiment was to investigate the effect of xanthan gum on the gel formation and properties of SDEW protein powder.

#### Single factor test of pH

Under the conditions of adding 0.08% xanthan gum, heating temperature 100°C, and heating time 1 h, six groups of different pH (5.0, 5.5, 6.0, 6.5, 7.0, and 7.5) were selected as the research objects. The objective of this experiment was to investigate the effect of pH on the gel formation and properties of SDEW protein powder.

#### Single factor test of heating time

Six groups of different heating times (20, 40, 60, 80, 100, and 120 min) were selected as the research objects under the conditions of xanthan gum dosage of 0.08%, pH 6.5, and heating temperature 100°C. The objective of this experiment was to investigate the effect of heating time on the gel formation and properties of SDEW protein powder.

#### Single factor test of heating temperature

Six groups of different heating temperatures (90, 95, 100, 105, 110, and 115°C) were selected as the research objects under the conditions of xanthan gum dosage of 0.08%, pH 6.5, and 1 h of heating time. The objective of this experiment was to investigate the effect of heating temperature on the gel formation and properties of SDEW protein powder.

### Measurement of gel strength

A cube (10 mm × 10 mm × 10 mm) was cut in the center of SEDW-XG gel (11). The gel strength of the gel was measured by TA. XT2i texture analyzer (TAXT2 of table Micro Systems, Ltd., Surrey, United Kingdom). The measurement parameters were as follows: the speed before the test was 2.0 mm/s, the speed during the test was 1 mm/s, and the speed after the test was 2 mm/s. The compression ratio of the texture analyzer was set to 50%, and the triggering force was set to 5.0 g. The hardness was recorded as gel strength in g/cm^2^.

### Measurement of color parameters

Color parameters of protein powder were determined by a chromatic aberration meter (CR-400, Konica Minolta Inc., Tokyo, Japan). Protein powders with different xanthan gum additions were placed in petri dishes and pressed vigorously. The flattened protein powder was placed under a chromatic meter to measure its L^*^, A^*^, and b^*^. The total color difference (ΔE) of protein powder samples was calculated using Equation 1 (16):


(1) $$\Delta E=\sqrt{{\left(L_{0}^{*}-L^{*}\right)}^{2}+{\left(a_{0}^{*}-a^{*}\right)}^{2}+{\left(b_{0}^{*}-b^{*}\right)}^{2}}$$

where the L_0_^*^, a_0_^*^, and b_0_^*^ indicate color parameter of protein powder sample.

### Measurement of water holding capacity

The determination of water holding capacity was based on the method of Jiang et al. (17). The gel sample (10 g) was weighed and denoted as M_1_. Then, the gel sample was put into the centrifuge tube for centrifugation (2,655 × *g*, 30 min). The water on the surface of the centrifuged gel sample was sucked up with filter paper, and the mass of the gel sample was measured, denoted as M_2_. The water holding capacity was calculated using Equation 2:


(2) $$WHC\;\left(\%\right)=\left(M_{2}/M_{1}\right)\times 100$$

### Observation of surface morphology of gel

The microstructure of the gel samples was observed and analyzed by referring to the method of Xu et al. (18). The gel samples were cut into a cube. The cut cubes were soaked in 2.5% glutaraldehyde solution for 12 h (4°C). The soaked gel samples were rinsed with phosphate buffer (0.02 mol/L, pH 7.4) at room temperature for 4 min. After rinsing, the gel samples were immersed in 50, 60, 70, 80, 90, and 100% ethanol solution successively (10 min each time). Then, the gel samples were soaked with isoamyl acetate for 15 min. The soaked samples were freeze-dried in a freeze-dryer. The freeze-dried samples were mounted on the scanning electron microscope screw root using a double-sided conductive adhesive. Then, the samples were coated with a thin layer of gold using a sputtering device. A scanning electron microscope (Quanta-200, FEI, Netherlands) was used to observe the microscopic morphology.

### Determination of Fourier transform infrared spectroscopy of gel

According to the method of Xu et al. (19), the gel samples were analyzed by Fourier transform infrared spectroscopy (IS10, Thermo Nicolet Corp., Madison, WI, United States). The gel samples were lyophilized. The freeze-dried sample (1 mg) was mixed with 100 mg of potassium bromide. The mixture was then ground into a uniform powder. The ground powder was pressed into thin wafers. The protein secondary structure of the gel was determined by an infrared spectrometer (FTIR, IS10, Thermo Nicolet Instruments, United States). The wave number range was 4,000 cm^−1^ ~ 400 cm^−1^, the resolution was 4 cm^−1^, the scanning times were 64, and the ambient temperature was 25°C. OMNICV8.0 software (Nicolet, United States) was used to analyze the spectral data. The relative percentages of secondary structures of different proteins were calculated according to the integrated peak area.

### Statistical analysis

The SPSS 20.0 software (IBM, Chicago, IL, United States) was used for the ANOVA of the samples in the study. Significant differences were determined by Duncan’s multiple comparison test (*p* < 0.05).

## Results and discussion

### Test results of xanthan gum content

**Figure 1:**
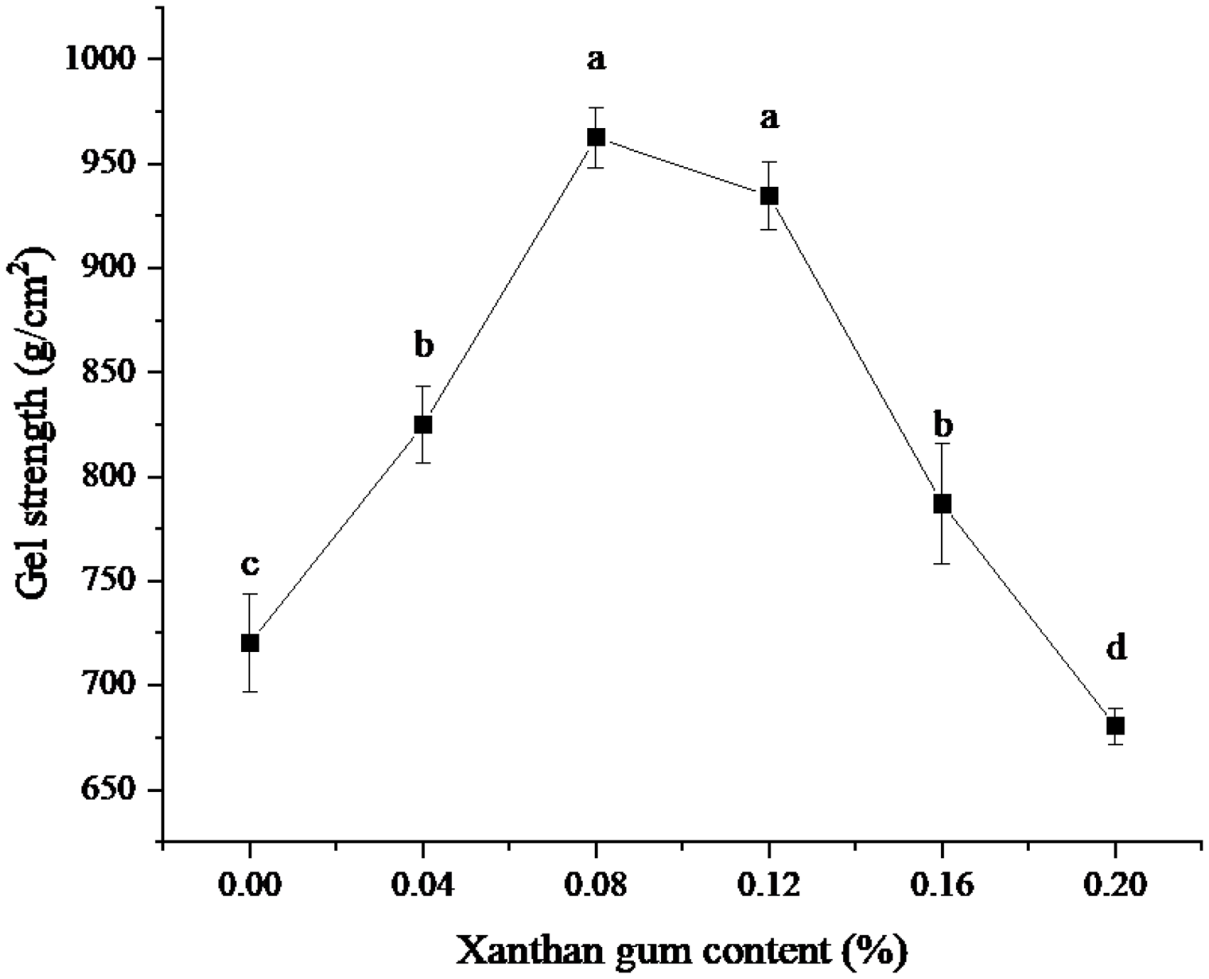
Effect of xanthan gum content on gel strength of protein powder. Different lowercase letters express the significant differences (*p* < 0.05).

The effects of different xanthan gum content on gel strength were shown in Figure 1. As can be seen from the figure, when the amount of xanthan gum was less than 0.08%, the gel strength kept increasing. When the amount of xanthan gum was 0.08%, the gel strength of the protein powder was larger. Subsequently, the gel strength of protein powder decreased gradually when the xanthan gum dosage was greater than 0.08%.

Gel strength is one of the important indexes of protein powder gel (20). The three-dimensional network structure formed by proteins is an important factor in maintaining gel properties. Adding material is one of the important means to improve the gel strength of SDEW protein powder (21). Xanthan gum is a kind of hydrophilic colloid, which belongs to polysaccharide. Xanthan gum is composed of D-glucose, D-mannose, and D-glucuronic acid. Appropriate amounts of xanthan gum can co-gel with protein and significantly improve the gel strength of protein powder. This may be since the lower amount of Xanthan gum can co-gel with proteins through electrostatic interaction and promote the formation of a three-dimensional network structure. However, a high amount of xanthan gum will generate electrostatic repulsion with protein, which is not conducive to the formation of gel (22).

### Test results of different pH

**Figure 2:**
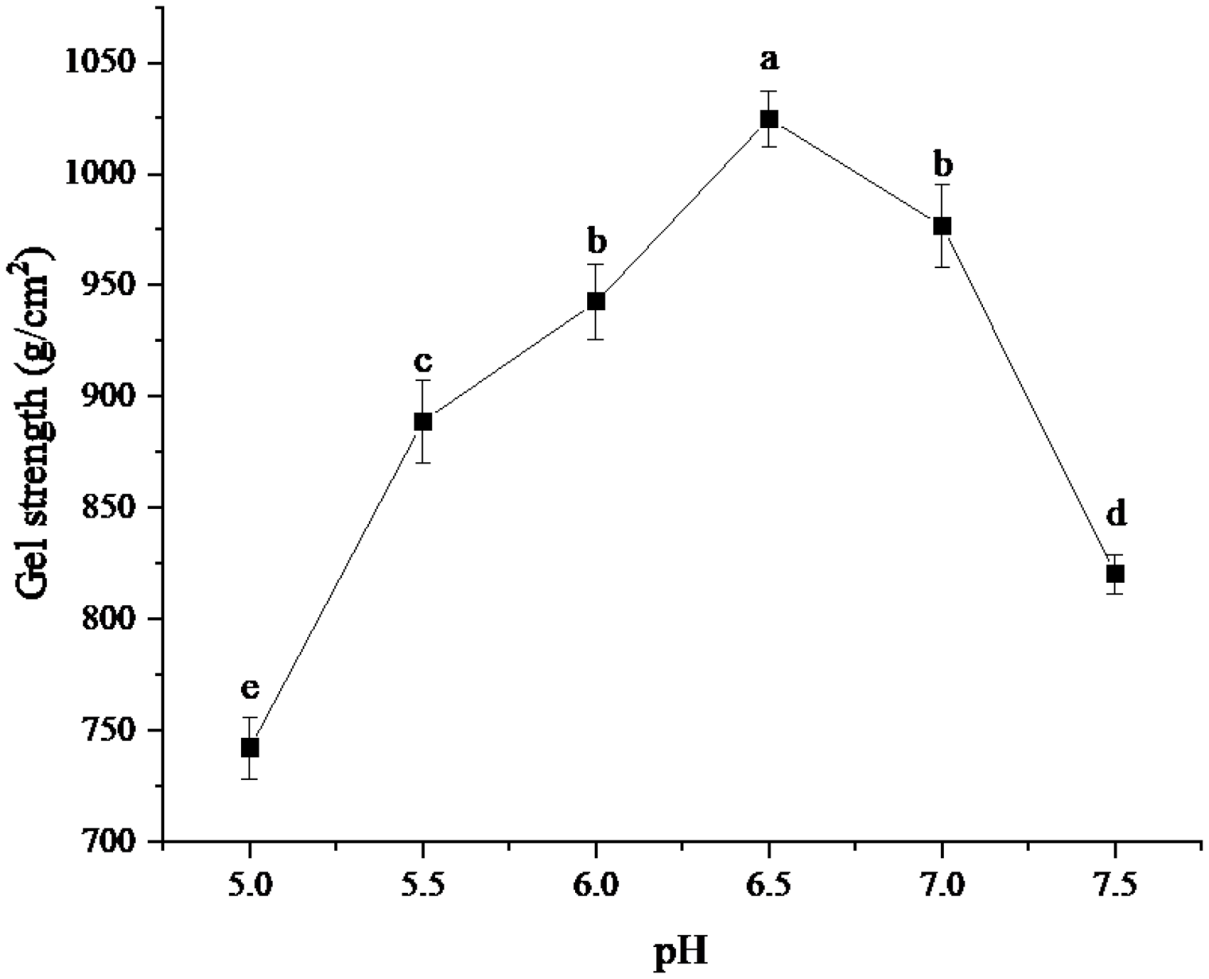
Effect of pH on gel strength of protein powder. Different lowercase letters express the significant differences (*p* < 0.05).

The effects of different pH on the gel strength of protein powder were shown in Figure 2. As can be seen from the figure, the gel strength of the protein powder increased with the increase in pH. When pH was 6.5, the gel strength of protein powder reached the maximum, about 1024.78 g/cm^2^. With the further increase of pH, the gel strength of protein powder gradually decreased. When pH was 7.5, the gel strength showed a sharp decline.

The change of gel strength of protein powder may be due to the break of hydrogen bonds in xanthan gum in an acidic or alkaline medium, which leads to the failure of co-gel interaction with egg albumin in SDEW, thus affecting the formation of three-dimensional network structure (23). The change in the gel strength of protein powder may also be related to the isoelectric point of ovalbumin. When the pH of the protein powder gel is far away from its isoelectric point, the electrostatic repulsion will be larger, and it is easier to form linear aggregation. However, when the pH of the protein powder gel is close to its isoelectric point, the charge on the protein surface is small or even shielded, which is more likely to form a disordered cluster structure (24).

### Test results of different heating temperature

**Figure 3:**
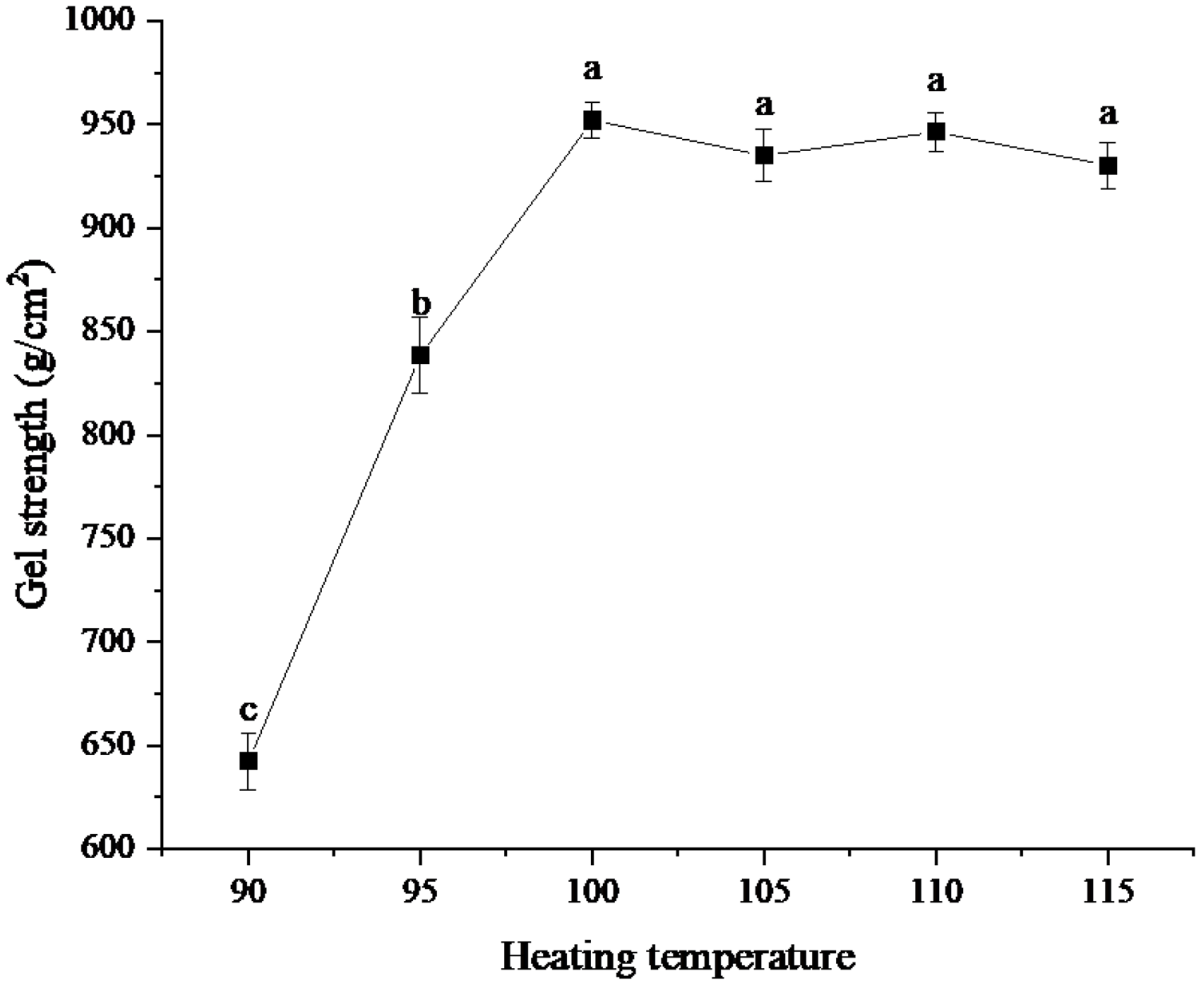
Effect of heating temperatures on gel strength of protein powder. Different lowercase letters express the significant differences (*p* < 0.05).

The effects of different heating temperatures on the gel strength of protein powder were shown in Figure 3. As can be seen from the figure, when the heating temperature was 90°C, the gel strength of protein was the lowest, about 642.36 g/cm^2^. Then, with the gradual increase in temperature, the gel strength of the protein powder increased rapidly. When the temperature reached 100°C, the gel strength was about 952.06 g/cm^2^. When the temperature exceeded 100°C, the gel strength was close to 950 g/cm^2^, but fluctuates slightly, and there was no significant difference.

The change in gel strength of protein powder with heating temperature may be because the protein molecular structure becomes more extended with increasing temperature, exposing more hydrophobic groups, which is conducive to the formation of gel (25). In the range below 100°C, the increase in temperature can promote the binding between protein molecules, form a larger network structure, and increase the gel strength. When the temperature rises above 100°C, protein molecules begin to degrade and destroy the intermolecular network structure, thus reducing the gel strength (26).

### Test results of different heating time

**Figure 4:**
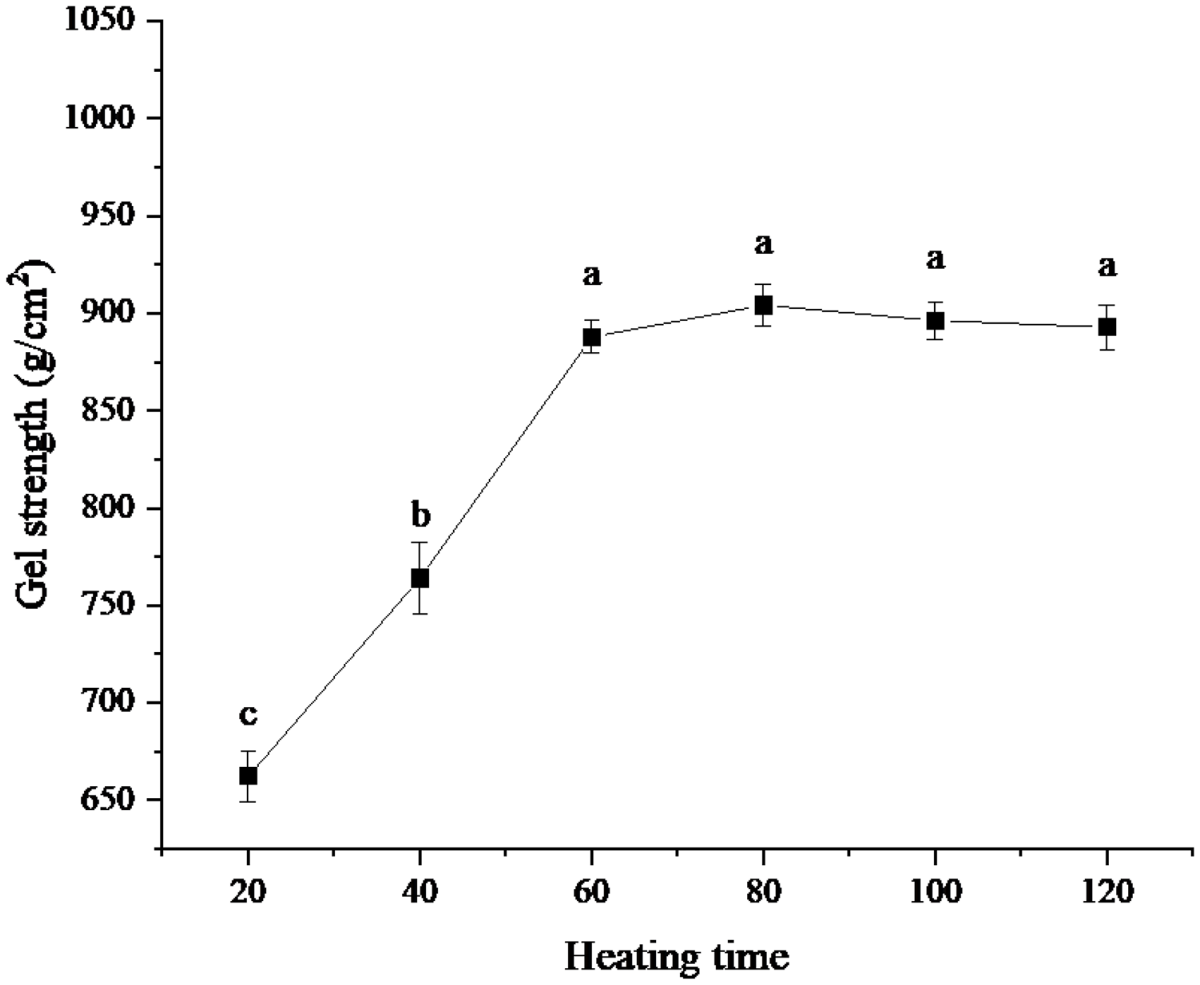
Effect of heating time on gel strength of protein powder. Different lowercase letters express the significant differences (*p* < 0.05).

The influence of different heating times on the gel strength of protein powder was shown in Figure 4. It can be seen from the figure that the gel strength of the protein powder reached its maximum when the heating time was 80 min. However, within the range of 60–120 min of heating time, there was no significant difference in gel strength among samples. When the heating time was less than 60 min, the gel strength of the protein powder was low. When the heating time was 20 min, the gel strength of the protein powder was the lowest.

When the heating time was less than 60 min, the gel strength of protein powder was low because xanthan gum cannot fully combine with protein to form SEDW-XG composite gel in a short time (27). However, when the heating time exceeded 60 min, the combination of xanthan gum and protein reached a saturation state due to the limited amount of xanthan gum and protein, so the gel strength of protein powder no longer changes (28).

### Optimization of experimental conditions using the single-factor test

Based on the discussion of xanthan gum addition amount, reaction pH, heating temperature, and heating time four single factors, the quadratic regression rotation design was carried out. Then, the single-factor test was carried out with the additional amount of xanthan gum, reaction pH, and heating temperature as independent variables, which had a great influence on the experimental results. Table 1 showed the results of the single-factor test with gel strength as the response value. Quadratic multivariate regression fitting was performed on the experimental data, and the analysis results showed that the regression equation of gel strength of SDEW protein powder was as follows: Y = 1037.00 + 21.88 A−3.12 B + 69.50C + 1.00 AB + 10.75 AC + 2.75 BC−80.25 A^2^−63.25 B^2^−122.50 C^2^ (A: Xanthan gum content (%); B: pH; C: Temperature; D: Time). Variance analysis was performed on the fitted quadratic polynomial, and the results were shown in Table 2.

**Table 1:** Results of single-factor experiment.

Groups	A: Xanthan gum content (%)	B: pH	C: Temperature (°C)	Gel strength (g/cm^2^)
1	0.08	6.5	105	864
2	0.08	6	100	868
3	0.08	6.5	95	763
4	0.08	7	100	873
5	0.1	6	95	786
6	0.1	6.5	100	1,032
7	0.1	7	95	761
8	0.1	7	105	922
9	0.1	6.5	100	1,042
10	0.1	6	105	936
11	0.1	6.5	100	1,020
12	0.1	6.5	100	1,023
13	0.1	6.5	100	1,086
14	0.12	6.5	105	927
15	0.12	7	100	921
16	0.12	6.5	95	783
17	0.12	6	100	912

**Table 2:** Analysis of variance on the results obtained using single-factor experiment.

Source	Square sum	Degrees of freedom	Mean square	*F*-value	*p*-Value	Significance
Model	1.615E+005	9	17942.30	52.29	<0.0001	**
A	3828.13	1	3828.13	11.16	0.0124	*
B	78.13	1	78.13	0.23	0.0467	*
C	38642.00	1	38642.00	112.62	<0.0001	**
AB	4.00	1	4.00	0.012	0.9170	
AC	462.25	1	462.25	1.35	0.2838	
BC	30.25	1	30.25	0.088	0.7751	
A^2^	27116.05	1	27116.05	79.03	<0.0001	**
B^2^	16844.47	1	16844.47	49.09	0.0002	**
C^2^	63184.21	1	63184.21	184.15	<0.0001	**
Error	2401.75	7	343.11			
Lack of fit	905.75	3	301.92	0.81	0.5520	
Net error	1496.00	4	374.00			
Total error	1.639E+005	16				

*Represents a significant difference (*p* < 0.05); **Represents a significant difference (*p* < 0.01).

It can be seen from the Table 2 that A, B, C, A^2^, B^2^, and C^2^ have a significant influence on the gel strength of protein powder. The high *F* value was 52.29, and the *p* value is <0.0001. The results showed that the fitting model could well explain the gel strength of SDEW protein powder. The *F* value and *p* value of the lack of fit were 0.81 and 0.0552 respectively, indicating that the predicted value of the model has high accuracy. Single-factor test showed that the optimum conditions of gel strength of SDEW protein powder were as follows: the content of xanthan gum was 0.08%, the reaction pH was 6.50, and the heating temperature was 100°C.

### Effect of xanthan gum content on the color of protein powder gel

As one of the important sensory indexes of food, color directly affects the quality of the high gelatinous salted duck egg protein powder (29). The results of gel color difference determination of SDEW protein powder with different xanthan gum contents were shown in Table 3. It can be intuitively seen from the table that the brightness of SDW was larger when the content of xanthan gum was 0.08 and 0.12. The redness and yellowness of SDW did not change significantly with the increase of xanthan gum content. The total color difference (∆E) between the groups was significant. It can be seen from the experimental results that the addition of xanthan gum causes changes in the gel brightness and total color difference of protein powder. However, the addition of xanthan gum had little effect on the redness and yellow color of the protein powder gel.

**Table 3:** Color parameters of protein powder gel with different xanthan gum contents.

Xanthan gum content (%)	L*	a*	b*	∆E
0.00	31.23 ± 2.49^ab^	0.23 ± 0.12^a^	3.53 ± 0.15^a^	–
0.04	30.67 ± 1.10^ab^	0.20 ± 0.08^a^	3.57 ± 0.19^a^	0.56 ± 0.05^e^
0.08	32.90 ± 1.28^a^	0.27 ± 0.09^a^	3.43 ± 0.21^a^	1.67 ± 0.11^c^
0.12	32.20 ± 1.15^a^	0.23 ± 0.12^a^	3.87 ± 0.42^a^	1.03 ± 0.09^d^
0.16	27.43 ± 3.29^b^	0.27 ± 0.05^a^	3.50 ± 0.25^a^	3.80 ± 0.17^a^
0.20	28.50 ± 0.94^b^	0.33 ± 0.05^a^	3.67 ± 0.15^a^	2.74 ± 0.17^b^

Results are mean ± standard deviation. Different lowercase letters in the same column express the significant differences (*p* < 0.05).

### Effect of xanthan gum content on water holding capacity of protein powder gel

**Figure 5:**
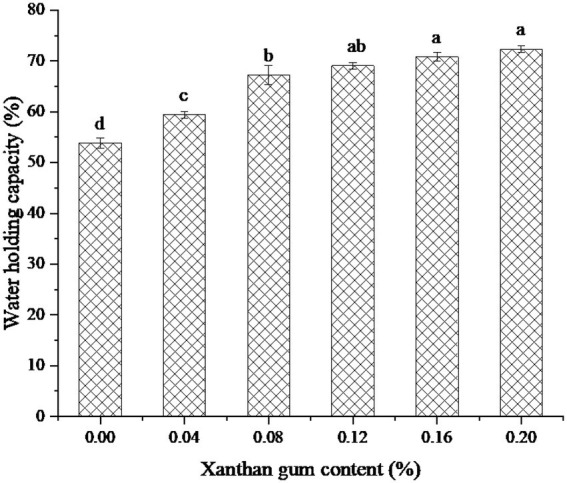
The water holding capacity of protein powder gel for xanthan gum addition. Different lowercase letters express the significant differences (*p* < 0.05).

Water holding capacity is one of the important functional properties of heat-induced protein gels. The water holding capacity is related to the pore size of the gel network and the properties of the biopolymer used (30). Figure 5 showed the water holding capacity of SEDW gel with different xanthan gum contents. As can be seen from the figure, SEDW gel containing 0.20% xanthan gum showed the best water holding capacity. However, the water holding capacity of SEDW gel did not increase significantly after the content of xanthan gum reached 0.08%. The possible reason was that the addition of xanthan gum can form a network structure with the macromolecules in SEDW gel and improve the water holding capacity of the gel. However, when the binding reached saturation, the water holding capacity of SEDW gel no longer increased (31).

### Effect of xanthan gum content on microstructure of protein powder gel

**Figure 6:**
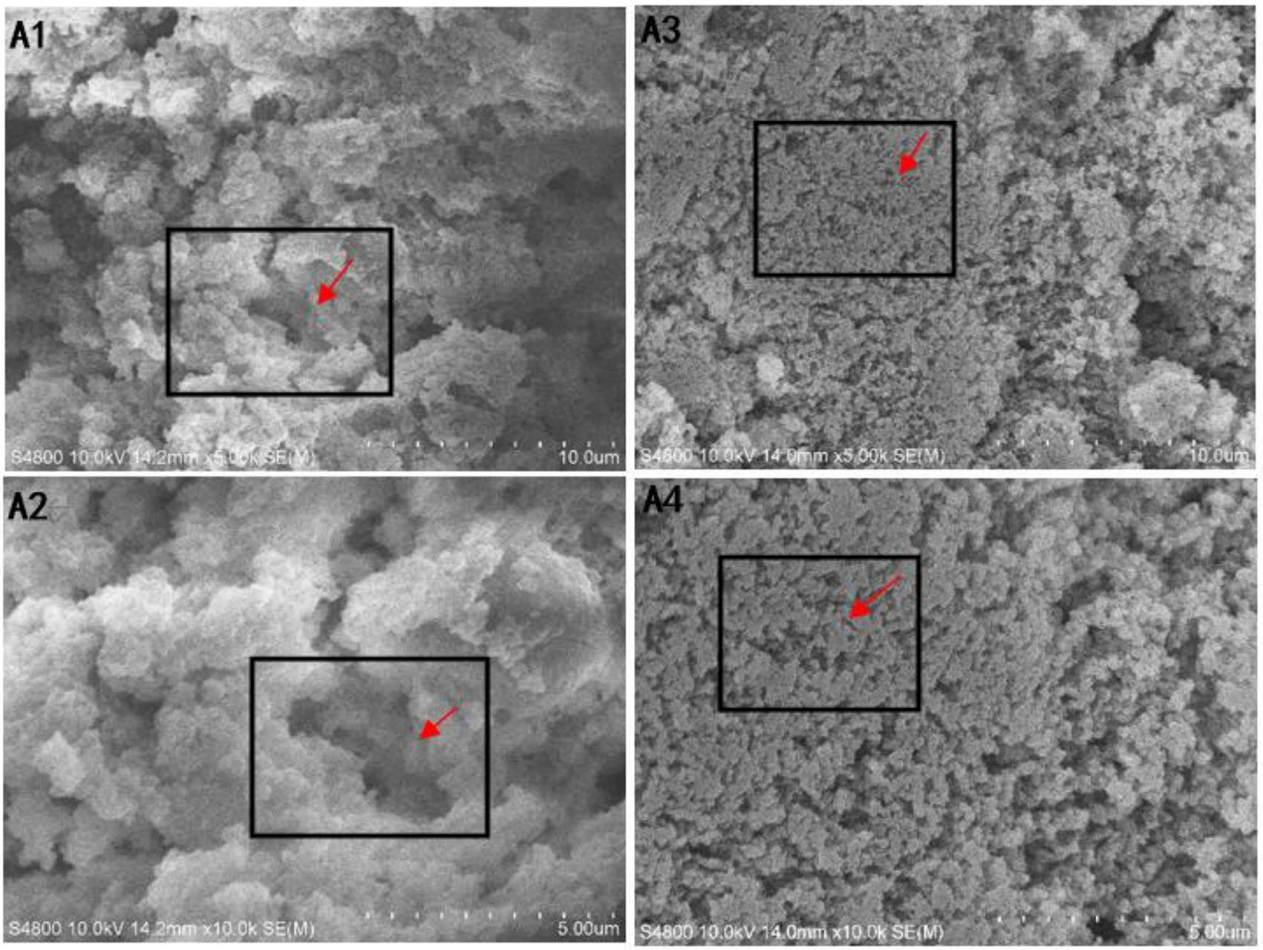
Comparison of gel microstructure between SDEW and SDEW-XG. A1: Gel made from SDEW (5.0 k magnification); A2: Gel made from SDEW (10.0 k magnification); A3: Gel made from SDEW-XG (5.0 k magnification); A4: Gel made from SDEW-XG (10.0 k magnification).

The microstructure of the thermally induced SDEW-XG gel was observed by scanning electron microscope. The following figures were from sample group (A1, A2): SDEW gel prepared without xanthan gum; Experimental group (A3, A4): SDEW-XG gel prepared under optimal conditions obtained by response surface analysis (Figure 6). As can be seen from the figure, the addition of xanthan gum can significantly improve the microstructure of the gel formed by protein powder. From the enlarged figure of 5.0 k, it can be seen that the surface microstructure of SDEW protein powder without xanthan gum was uneven, and the number of holes was very large. After xanthan gum was added, the gel microstructure particle size was more uniform, the surface was smoother and tighter, and the number of pores was significantly reduced. From the enlarged figure of 10.0 k, it can be more intuitively observed that the pores in the microstructure of SDEW protein powder without xanthan gum were of different sizes and generally had larger pore sizes. However, after the addition of xanthan gum, although there were holes in the gel microstructure, the size was uniform and the pore size was generally small. According to the experimental data, the gel strength of the experimental group was significantly higher than that of the control group. Therefore, the microstructure of the gel network can also be considered an important factor affecting the gel strength of protein powder. Protein powder gel with a more compact structure has higher strength (32).

### Effect of xanthan gum content on the secondary structure of protein powder gel

**Figure 7:**
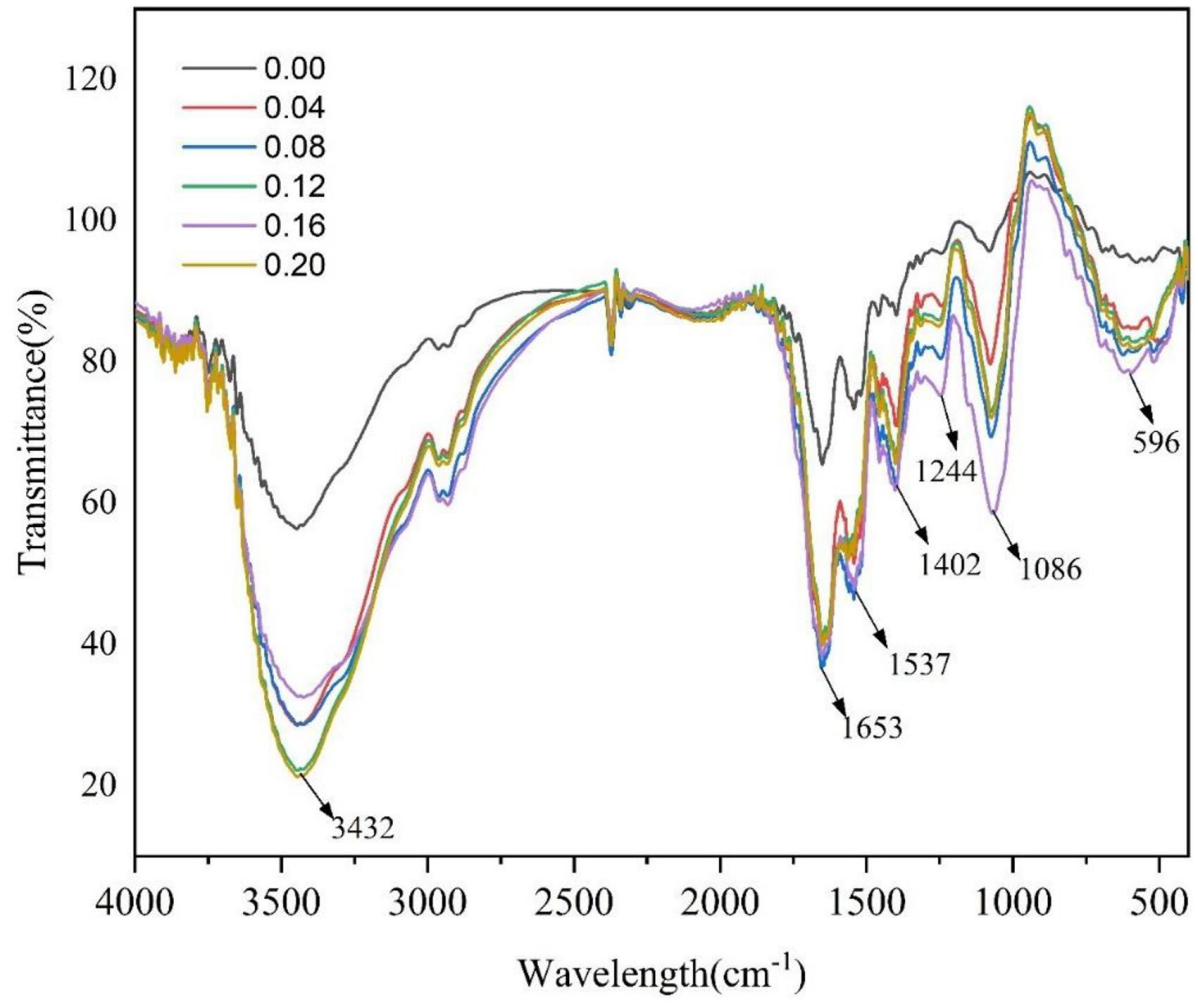
Effect of xanthan gum content on secondary structure of protein powder.

Proteins can form specific secondary structures, including α-helix, β-folding, and random curling (33). The amide I region band (1,600–1,700 cm^−1^) in the infrared spectrum is related to C=O tensile vibration, and can be used to evaluate the changes in secondary structure in proteins (34). As can be seen from Figure 7 and Table 4, the addition of xanthan gum significantly affected the secondary structure of SDEW protein powder. With the increase of xanthan gum content, β-fold content increased and α-helix content decreased. In the secondary structure of proteins, α-helix and β-folding are ordered, β-turning is a relatively loose partial order, and random curling is loose and disordered. According to the analysis of the peak value in the figure and the data in the table, the addition of xanthan gum changed the original rigid structure of the gel formed by egg white protein powder, leading to an increase in flexibility (35).

**Table 4:** Estimated secondary structure using the amide I region of SDEW protein powder.

Xanthan gum content (%)	α-helix%	β-sheet%	β-turn%	Random coil%
0.00	29.07 ± 0.35^a^	43.67 ± 1.68^b^	15.57 ± 0.41^b^	11.25 ± 0.38^b^
0.04	28.61 ± 0.52^a^	44.35 ± 1.57^b^	15.67 ± 0.38^b^	11.08 ± 0.47^b^
0.08	24.35 ± 0.92^b^	46.85 ± 2.37^ab^	16.09 ± 0.56^b^	12.53 ± 0.53^a^
0.12	22.48 ± 1.08^b^	47.21 ± 1.95^ab^	18.36 ± 1.23^a^	11.86 ± 0.96^ab^
0.16	20.95 ± 0.69^bc^	48.64 ± 2.08^a^	18.94 ± 1.68^a^	11.29 ± 0.82^ab^
0.20	18.36 ± 0.84^c^	49.63 ± 3.16^a^	19.07 ± 1.38^a^	12.60 ± 0.51^a^

Results are mean ± standard deviation. Different lowercase letters in the same column express the significant differences (*p* < 0.05).

## Conclusion

In this work, xanthan gum was added to study the high gelation of salted duck egg protein powder. The results showed that the addition of xanthan gum significantly improved the gel properties of salted duck egg protein powder. Compared with the ordinary protein powder, the gel property of the protein powder was greatly improved, and its water holding capacity was also on the rise. Secondly, the gel microstructure of high-gelatinous SDEW protein powder was smoother and tighter. The number of holes in the 3D network structure of the gel was also small, and the pore size was relatively uniform. The research of xanthan gum on the gelation enhancement mechanism of salted duck egg protein powder was not deep enough. In addition, the high gel egg white protein powder is mainly used in industry, and there is still a lot of work to be done in its development and industrial large-scale production.

## Data availability statement

The original contributions presented in the study are included in the article/supplementary material, further inquiries can be directed to the corresponding authors.

## Author contributions

XY, TD, YX, and MH: experiments and writing – original draft. JX, and JG: writing – review and editing. All authors have read and agreed to the published version of the manuscript.

## Funding

The authors acknowledged the support of the Anhui Provincial Science and Technology Major Special Project (202003b06020004), the Anhui Provincial Natural Science Foundation (No. 1908085MC79), and the Overseas Visiting and Study Program for Outstanding Young Backbone Talents of Anhui Universities (No. gxgwfx2020056).

## Conflict of interest

The authors declare that the research was conducted in the absence of any commercial or financial relationships that could be construed as a potential conflict of interest.

## Publisher’s note

All claims expressed in this article are solely those of the authors and do not necessarily represent those of their affiliated organizations, or those of the publisher, the editors and the reviewers. Any product that may be evaluated in this article, or claim that may be made by its manufacturer, is not guaranteed or endorsed by the publisher.
